# Improvement of shelf life of soymilk using immobilized protease of *Oerskovia xanthineolytica* NCIM 2839

**DOI:** 10.1007/s13205-016-0479-6

**Published:** 2016-08-08

**Authors:** A. K. Sahoo, V. S. Gaikwad, R. C. Ranveer, P. B. Dandge, S. R. Waghmare

**Affiliations:** 1 Department of Food Science and Technology, Shivaji University, Vidyanagar, Kolhapur, 416004 India; 2 Department of Biochemistry, Shivaji University, Vidyanagar, Kolhapur, 416004 India; 3 Department of Microbiology, Shivaji University, Vidyanagar, Kolhapur, 416004 India

**Keywords:** Protease, Soymilk, Immobilization, *Oerskovia xanthineolytica* NCIM 2839

## Abstract

Protease enzyme has lot of commercial applications, so the cost-effective production of protease using sunflower oil seed waste was carried out from *Oerskovia xanthineolyitca* NCIM 2839. The maximum protease production was after 24 h of incubation with 2.5 % oil seed waste concentration. *O. xanthineolytica* was found to produce two proteases—P1 and P2. The proteases were purified using 60 % cold acetone precipitation and DEAE-cellulose ion exchange chromatography. SDS-PAGE revealed molecular weight of P1 and P2 was 36 and 24 kDa, respectively. P1 and P2 were optimally active at pH 7.0 and pH 7.5 at temperature 35 and 40 °C, respectively. Analysis of hydrolyzed product of P1 and P2 by HPLC reveals that the P1 has endoprotease and P2 has exoprotease activity. The treated soy milk with immobilized proteases showed increased shelf life and removal of off flavor.

## Introduction

Proteolytic enzymes constitute one of the most important groups of commercial enzymes. These enzymes have ample utilization in industrial processes, such as the detergent industry, a major consumer of proteases, as well as food and leather industries (Kumar and Takagi 1999; Gupta et al. 2002). Proteases are ubiquitous enzymes occurring in wide diversity of species including plants, animals and microorganisms. The vast range led to numerous attempts to exploit their biotechnological applications and established proteases as one of the major groups of industrial enzymes (Rao et al. 1998). Proteases are involved in numerous biological functions, such as septum formation, sporulation, protein turnover, catabolite inactivation, protein secretion and nutrition (Van Tilburg et al. 1984; Godfrey and West 1996).

Microorganisms are the most preferred source of these enzymes in fermentation bioprocesses not only because of their fast growth rate, but also for their ability to engineer genetically to generate new enzymes with desirable abilities or simply for enzyme overproduction (Rao et al. 1998; North 1982). Microbial proteases play an important role in biotechnological processes and they account for approximately 59 % of the total enzymes used (Spinosa et al. 2000). Proteases are produced by wide range of microorganisms including bacteria, molds, and yeasts. Among bacteria, the genus *Bacillus* predominantly produces extracellular proteases (Godfrey and Reichelt 1985). *O. xanthineolytica* was potentially known for its ability to produce alkaline protease (Saeki et al. 1994) and thermotolerant chitinase (Waghmare and Ghosh 2010).

Protease production depends on many factors, such as the growth rate of the culture and the composition of the medium plays important roles (Johnvesly et al. 2002). Carbon and nitrogen sources at high concentration were considered detrimental factors in protease production (Frankena et al. 1986). Several studies had reported that proteins and peptides were necessary for effective protease production (Drucker 1972). Some work reported better protease synthesis in the presence of glucose as a carbon source (Gessesse and Gashe 1997). Other medium compounds, such as metal ions and phosphorous source, may also affect the amount of enzyme formation. Several reports have suggested that proteins from the food stuffs hydrolyzed by proteolytic enzymes lead to formation of bioactive peptides (Maestri et al. 2016; Mora et al. 2016; Moughan and Rutherfurd-Markwick 2013). In this study, attempt has been made for the economic production of protease from *Oerskovia xanthineolytica* NCIM 2839 using oil industry solid waste and immobilized protease used for the improvement of soymilk quality such as flavor and shelf life.

## Materials and methods

### Microorganism and culture conditions

*Oerskovia xanthineolytica* NCIM 2839 was obtained from the National Collection of Industrial Microorganisms (NCIM), Pune, India. The culture was maintained on nutrient agar (Peptone 1.0 %, Beef extract 1.0 %, NaCl 0.5 %, Agar powder 1.5 %) at 4 °C and subcultured after every 15 days.

### Analysis of sunflower oil industry solid waste

Chemical analysis of waste was carried out to determine protein, fat, moisture, ash and total sugar using standard protocol described by Egan et al. (1981). The protein content was determined by microKjeldahl method, moisture content by oven drying method, fat content by Soxhlet method, total sugar by phenol–H_2_SO_4_ method.

### Protease production

The protease production was carried out by submerged fermentation technique. Medium used for the protease production contains K_2_HPO_4_ 0.1 %, MgSO_4_ 0.05 %, FeSO_4_ 0.001 %, sunflower oil solid waste 2.5 % and distilled water 100 ml (pH 7.0). The 24-h-old fresh culture of *O. xanthineolytica* NCIM 2839 was inoculated in conical flask containing 100 ml medium. The flasks were incubated at 30 °C and protease activity was monitored after every 6-h interval for 30 h. To study the effect of waste concentration on production of protease in the medium, the production was carried out at various concentrations of waste such as 0.5, 1.0, 1.5, 2.0, 2.5, and 3.0 %.

### Purification of proteases

All steps of purification were carried out at 4 °C. The growth of *O. xanthineolytica* was inoculated in medium with 2.5 % sunflower oil industry solid waste. After 24 h incubation at 30 °C, the broth was centrifuged at 2795×*g* for 20 min to obtain cell-free medium. This was then subjected to precipitation using 50 % cold acetone. The precipitate was collected by centrifugation at 11,180×*g* for 30 min, dissolved in 25 mM sodium phosphate buffer of pH 7.0 and dialysed against the same buffer. The enzyme was further purified by DEAE-cellulose column chromatography, and the column was eluted with NaCl gradient from 0.1 to 0.5 M concentrations, and fraction of 5 ml was collected at the flow rate of 1.0 ml min^−1^. All the fractions were checked for their protein content by the method of Lowry et al. (1951) and also for protease activity. The fractions showing protease activity were used further for characterization.

### SDS-PAGE analysis

Purity of the fractions, showing protease activity, was checked by SDS-PAGE by the method of Laemmli (1970). The bands were visualized by silver staining technique (Merril 1987). The molecular weight of proteases was determined by comparison with standard molecular marker proteins (Phosphorylase b 98 kDa, bovine serum albumin 66 kDa, Ovalbumin 43 kDa, carbonic anhydrase 29 kDa, Soyabean Trypsin inhibitor 20 kDa).

### Enzyme assay

In the protease activity assay, P1 and P2 enzymes (0.5 ml) were mixed with 2.5 ml of 0.5 % casein in sodium phosphate buffer (pH 7) and incubated at 30 °C for 10 min. The reaction was terminated by adding 5 ml of 0.19 M trichloroacetic acid (TCA). The reaction mixture was centrifuged and resultant soluble peptide of supernatant was measured with tyrosine as the reference substance (Liang et al. 2006). One unit of protease activity is defined as the activity that releases 1 µmol of tyrosine in 1 min at specified conditions.

### Effect of pH and temperature on protease activity

The effect of pH on enzyme activity of P1 and P2 protease was studied at various pH ranging from 3.0 to 10.0 using different buffer systems. The buffers used for the purpose were 25 mM citrate (pH 3.0–5.0), 25 mM sodium phosphate (pH 6.0–8.0), and 25 mM glycine–NaOH (pH 9.0–10). The optimum temperature for enzyme activity was determined by assaying residual enzyme activity at various temperatures from 10 to 80 °C. In the temperature stability study, the enzyme was kept for 24 h at the 10–70 °C and residual activity was measured.

### Effect of different metals ions on protease activity

The proteases P1 and P2 were pre-incubated in 25 mM sodium phosphate buffer having pH 7.0 and 7.5 for the respective enzymes along with metal ions Fe^3+^, Hg^2+^, Cu^2+^, Zn^2+^, Mg^2+^, Mn^2+^ using their respective water-soluble salts at 5 mM concentrations. The residual activities of P1 and P2 were checked by standard assay as mentioned above.

### Analysis of hydrolysed products

The purified P1 and P2 enzymes were separately incubated with casein, as stated in enzyme assay section. After a 10-min incubation period, products were analyzed by HPLC (Waters 2690 System) using C18 column (4.6 × 250 mm). Elution was done with 70 % acetonitrile at a flow rate of 1 ml min^−1^, which was monitored by measuring UV *A*_220_ with a Waters Lambda-Max model with LC Spectrophotometer.

### Immobilization of enzyme and use in soy milk preparation

The enzyme was immobilized in calcium alginate beads according to the method described by Ates and Mehmetoglu (1997); in this method, 8 ml of sodium alginate solution (3.75 %) was mixed with 2 ml of partially purified enzyme solution (10 mg ml^−1^) to form a homogenous final alginate concentration of 3 %. The mixture was extruded drop by drop into 0.2 M CaCl_2_ solution at 4 °C to form beads using a sterile hypodermic syringe needle. The beads were allowed to harden in the CaCl_2_ solution for 2 h. The resulting spherical beads were washed with sterile distilled water. The beads were stored in 25 mM sodium phosphate buffer (pH 7.0) at 4 °C. Soymilk was prepared according to the method described by Gatade et al. (2009). The prepared soymilk was treated with immobilized enzyme and incubated at 30 °C for 1 h. Then treated milk was compared with control milk (untreated) for organoleptic characteristics such as color, texture and flavor.

### Statistical analysis

Results obtained were the mean of three or more determinants. Analysis of variance was carried out on all data at *p* < 0.05 using Graph Pad software (GraphPad InStat version 3.00).

## Results and discussion

### Proximate analysis of sunflower oil seed waste

The sunflower oil seed waste used for the protease production was analyzed for protein, total sugar, fat, and ash content. The obtained results are shown in Table 1; sunflower oil seed waste was found to be rich in carbohydrates, protein, fat, and minerals, which can act as a good medium for the growth of microorganisms.

**Table 1:** Proximate composition of sunflower waste

Components	Amount (%)
Protein	48.55 ± 1.20
Total sugar	24.75 ± 0.90
Fat	16.20 ± 0.65
Moisture	09.20 ± 0.84
Ash	01.30 ± 0.65

Each value represents the mean ± standard error values

### Production of protease

**Fig. 1:**
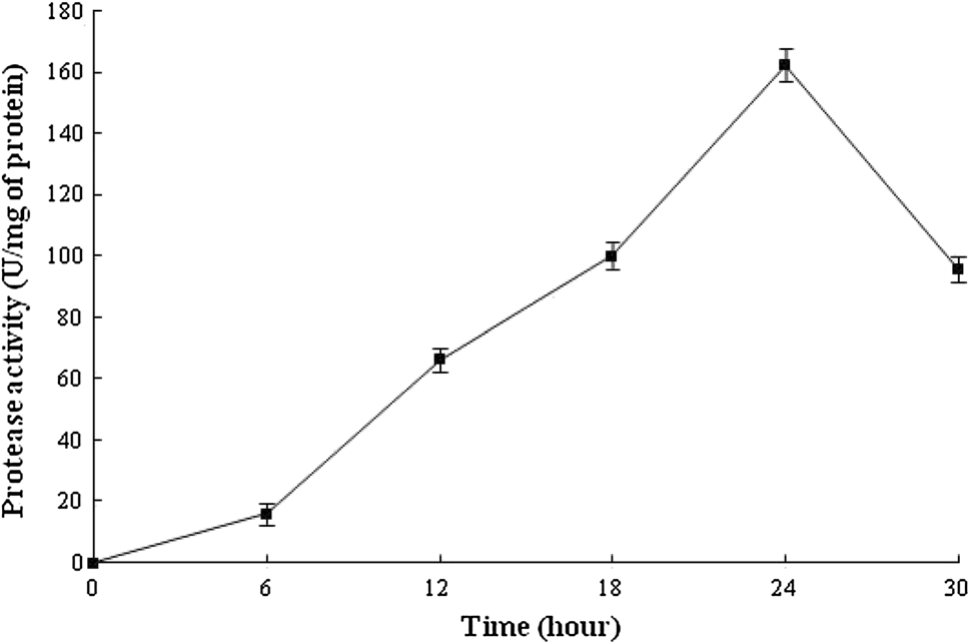
Protease production monitoring during incubation period. In this experiment, the protease activity was carried out by standard protease assay described in enzyme assay section

**Fig. 2:**
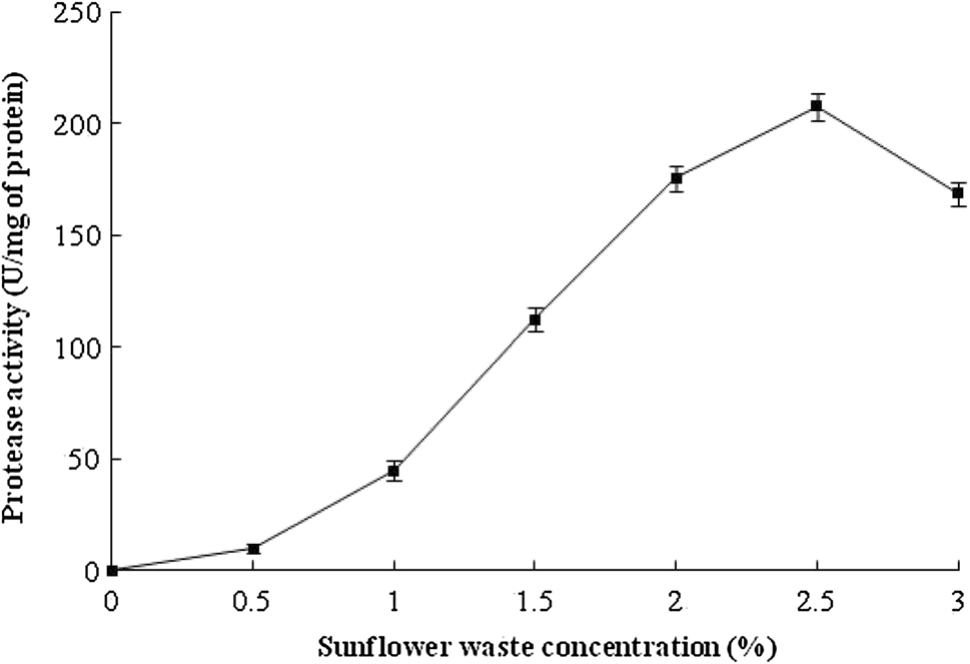
Effect of sunflower oil seed waste concentration on the production of protease from *O. xanthineolytica* NCIM 2839. The protease production was carried out in medium containing 0.5–3 % sunflower oil seed waste and protease activity was checked

*O. xanthineolytica* NCIM 2839 was found to produce extracellular proteolytic enzymes in the presence of sunflower oil seed waste as carbon and nitrogen source. Similarly, De Azeredo et al. (2006) have used feather meal and corn steep liquor for thermophilic protease production from *Streptomyces* sp. 594. The protease activity in supernatant was increased up to 24 h as incubation time increased; after 24 h, the activity decreased sharply, as shown in Fig. 1. It is vivid from Fig. 2 that maximum protease activity was in medium containing 2.5 % sunflower oil seed waste, and the protease production decreased as the concentration of sunflower oil seed waste increased 2.5–3.0 %.

### Purification of proteases

**Fig. 3:**
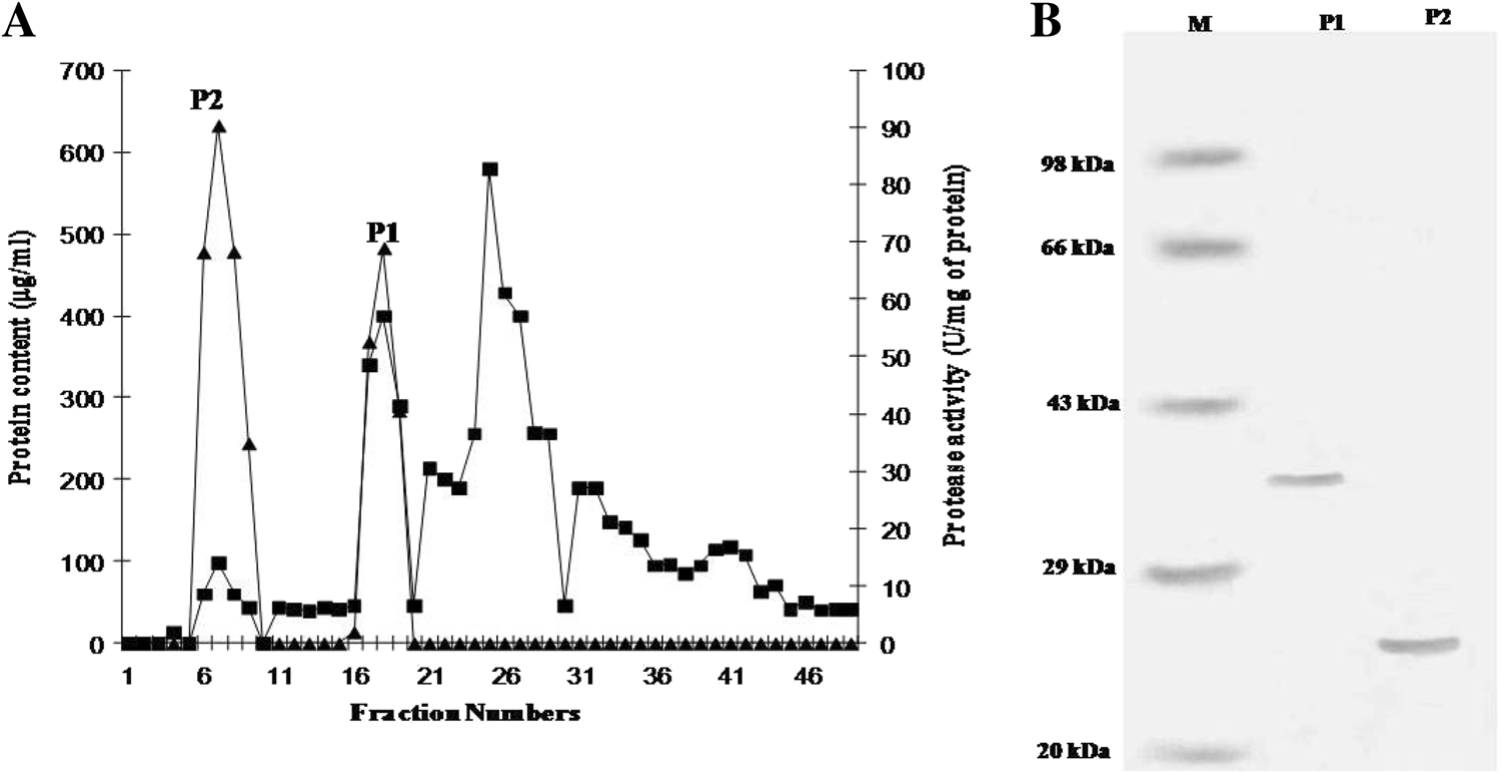
**a** Purification profile of P1 and P2 protease of *O. xanthineolytica* NCIM 2839. The proteases were purified using DEAE-Cellulose ion exchange column chromatography, fractions collected (*filled square*) and protease activity (*filled triangle*). **b** SDS-PAGE analysis of purified P1 and P2 proteases. *Lane M* is standard molecular weight marker proteins, *Lane P1* DEAE-Cellulose chromatography fraction of P1 protease, and *Lane P2* DEAE-Cellulose chromatography fraction of P2 protease

An effective scheme for protease purification was developed combining cold acetone precipitation and DEAE-cellulose ion exchange chromatography. The protease was precipitated from the cell-free medium optimally at 60 % cold acetone concentration and applied to the DEAE-cellulose column. There are many proteins eluted from 0.1 M NaCl to 0.5 M NaCl concentration as shown in the chromatogram, but only two peaks showed protease activity (P1 and P2), as shown in Fig. 3a. The P1 was eluted at 0.1 M NaCl concentration, whereas P2 eluted at 0.2 M NaCl concentration. The molecular weights of P1 and P2 were found to be 25 kDa and 36 kDa, respectively, on the SDS-PAGE, as shown in Fig. 3b, which is comparable to those reported from other microorganisms such as 45 kDa from *Bacillus subtilis megatherium* (Gerze et al. 2005) and 28 kDa from *Bacillus* sp. B16 (Qiuhong et al. 2006).

### Effect of pH and temperature on enzyme activity

**Fig. 4:**
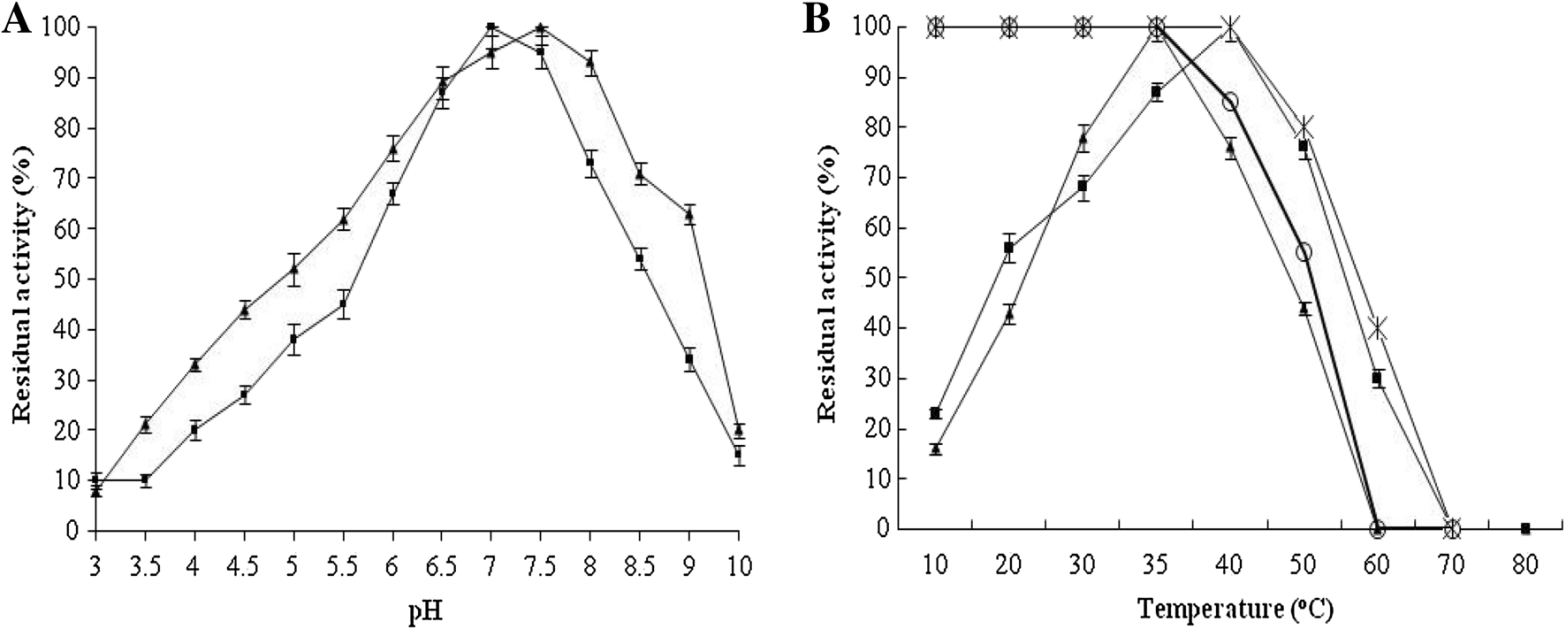
**a** Effect of pH on purified P1 and P2 proteases. Residual activity of purified P1 protease (*filled square*) and of purified P2 protease (*filled triangle*) at various pH. **b** Effect of temperature on purified P1 and P2 proteases and its stability. Residual activity of purified P1 protease (*filled square*) and of purified P2 protease (*filled triangle*) at various temperatures and stability of P1 (*multiple symbol*) and P2 (*open circle*)

Although the enzymes were active at broad range of pH, 4.0–9.0, optimum activity was observed at pH 7.0 and pH 7.5 of P1 and P2 enzyme, respectively, as shown in Fig. 4a. P1 and P2 enzymes were retaining their 50 % activity between pH 5.5 and 8.0; this indicates that the enzyme was neutrophilic in nature. Saeki et al. (1994) reported alkaline protease from *O. xanthineolytica* strain TK-1 which is optimally active at pH 9.5–11.0 at 50 °C. It was observed that the optimal temperature for enzyme activity was 35 and 40 °C of P1 and P2 enzyme, respectively, as shown in Fig. 4b, where both the enzymes retained their 50 % activity between temperature 30 and 50 °C. The P1 enzyme was found to be more stable than the P2 enzyme, as shown in Fig. 4b.

### Effect of metal ions on enzyme activity

The effect of metal ions on P1 and P2 proteases is summarized in Table 2. Mn^2+^ was found to be an activator of P1 and P2, whereas Mg^2+^ activates only P1. Enzyme activity was completely inhibited in the presence of Hg^2+^. Cu^2+^ was found to be an inhibitor of P2 and slightly inhibitory for P2, whereas no effect was observed in the presence of Fe^3+^ ions.

**Table 2:** Effect of metals ion on P1 and P2 protease activity

Metals ions	Residual activity (%)	
	P1 protease	P2 protease
	5 mM	5 mM
None	100.0	100.0
Zn^2+^	100.0	40.0
Hg^2+^	0.0	0.0
Mg^2+^	150.0	100.0
Mn^2+^	120.0	130.0
Fe^3+^	100.0	100.0
Cu^2+^	70.0	0.0

### Analysis of hydrolyzed products

**Fig. 5:**
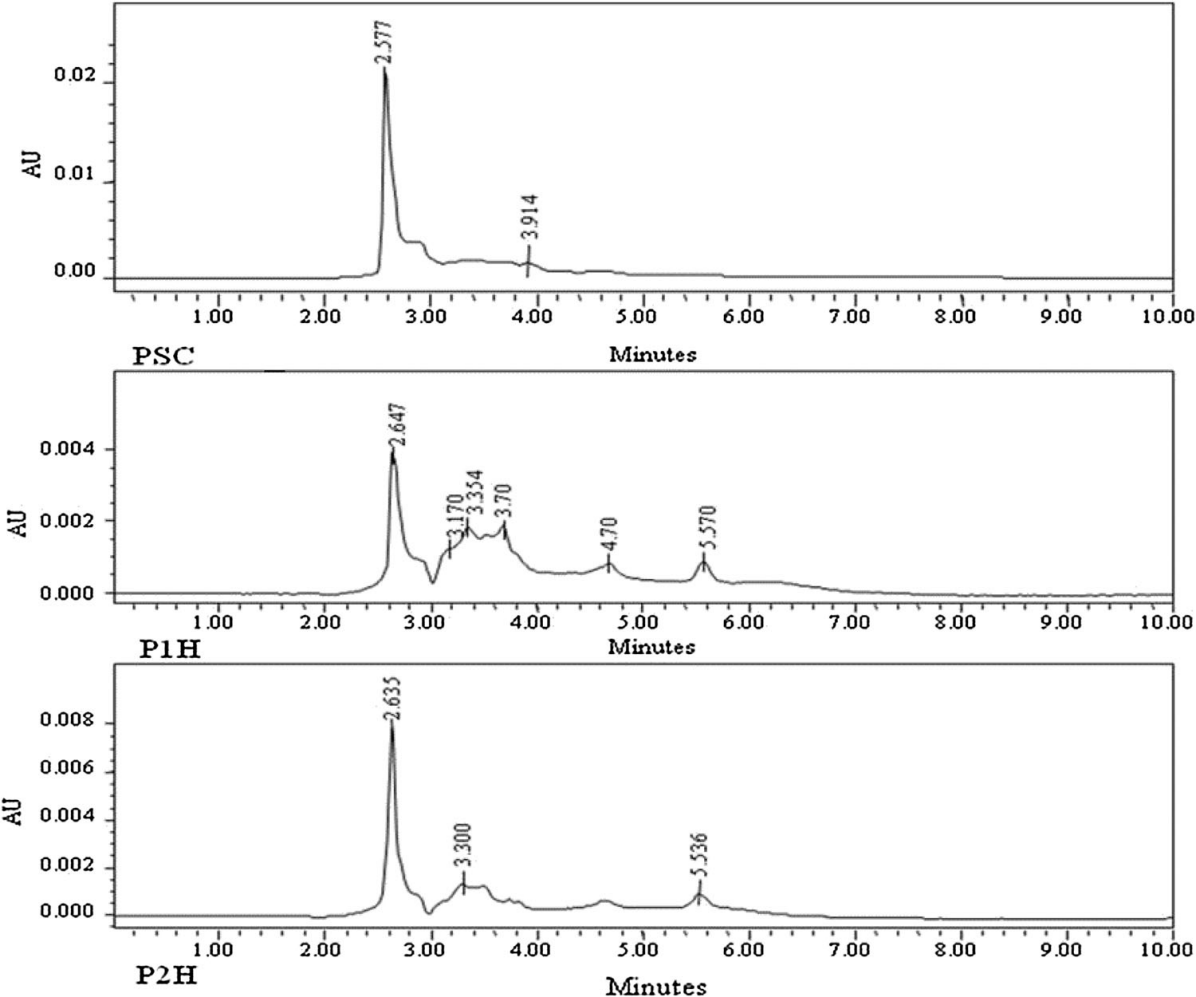
HPLC analysis of hydrolysed products of P1 and P2 proteases of *O. xanthineolytica* NCIM 2839. The purified protease P1 and P2 was incubated with casein at 30 °C for 10 min and hydrolyzed products were detected by HPLC. *PSC* substrate control, *P1H* products formed after action of P1 enzyme and *P2H* products formed after action of P2 enzyme

After casein was hydrolysed by P1 and P2 separately, the products were analyzed by HPLC as shown in Fig. 5. It was observed that P1 hydrolyses casein into oligopeptides (Fig. 5, P1H), whereas P2 hydrolyses into amino acids (Fig. 5, P2H). This indicates that *O. xanthineolytica* NCIM 2839 produces a protease system which has two components, i.e., P1 and P2, the P1 has endoprotease activity and P2 has exoprotease activity.

### Treatment of soymilk with immobilized protease

Protease immobilized by the Ca-alginate method was found to be more stable than the free enzyme. The earlier reports suggest that the protease can be immobilized using agar, sodium alginate, polyacrylamide and gelatin, but more leakage was observed with agar and less with gelatin (Kumar and Vats 2010). So Ca-alginate method could be preferable for immobilization of protease. After treatment of the soymilk using immobilized proteases, it was observed that the beany flavor of the soymilk was reduced and gives the pleasant smell which makes soymilk more acceptable for consumers. For a long time, it was known to human that amino acids which are produced by the action of protease give flavor to the product. It was also found that treated milk using immobilized proteases showed increased shelf life at 4 °C up to 15 days than the control which was up to 10 days. The shelf life could be increased because of antimicrobial peptides produced by the action of proteases of *O. xanthineolytica* NCIM 2839. Several reports on enzymatic hydrolysis of soy protein have promoted numerous bioactive functions, such as angiotensin-I converting enzyme inhibitory activity (Chiang et al. 2006), adipogenesis inhibitory activity (Tsou et al. 2010a, b), cholesterol-lowering activity (Tsou et al. 2009) and anti-oxidative activity (Moure et al. 2006).

## Conclusion

In the present study, the low-cost production of protease from *O. xanthineolytica* NCIM 2839 using sunflower oil seed waste was carried out. As far as we are concerned, our work is the first contribution toward the production of protease using sunflower oil seed waste from *O. xanthineolytica* NCIM 2839. After the treatment of soy milk with protease, increased shelf life of soy milk encourages possible application of protease for the food industries.
